# Thyroid disorders in Brazil: the contribution of the Brazilian Longitudinal Study of Adult Health (ELSA-Brasil)

**DOI:** 10.1590/1414-431X20198417

**Published:** 2019-02-14

**Authors:** I.M. Bensenor

**Affiliations:** 1 Centro de Pesquisa Clínica e Epidemiológica, Hospital Universitário, Universidade de São Paulo, São Paulo, SP, Brasil; 2 Departamento de Clínica Médica, Faculdade de Medicina, Universidade de São Paulo, São Paulo, SP, Brasil

**Keywords:** Subclinical thyroid diseases, Hyperthyroidism, Hypothyroidism, Subclinical atherosclerosis, Cardiovascular diseases, Mental health

## Abstract

Thyroid disorders are common diseases, both in Brazil and worldwide. The Brazilian Longitudinal Study of Adult Health (ELSA-Brasil) is a prospective cohort study that investigates cardiovascular diseases, diabetes, and associated factors, including non-classical cardiovascular risk factors such as thyroid function. Thyroid function was classified according to thyrotropin stimulating hormone (TSH), free thyroxine (FT4), and use of medication to treat thyroid disorders, after excluding participants who reported use of any medication that could alter the results of the TSH and FT4 tests. All analyses included in this review are cross-sectional using baseline data (2008 to 2010). The results showed an association of subclinical thyroid disorders with biomarkers of subclinical atherosclerosis, measured by carotid intima-media thickness and coronary artery calcium, insulin resistance, metabolic syndrome, and some psychiatric disorders. No association was found with the biomarker of inflammation high-sensitivity C-reactive protein, or changes in pulse wave velocity or heart rate variability.

## Introduction

The Brazilian Longitudinal Study of Adult Health (ELSA-Brasil) is a prospective cohort study with 15,105 civil servants from six cities in Brazil. The main objective of the study is to investigate the incidence of cardiovascular diseases and diabetes, and their associated factors (1–3). However, the study attempts to capture not only the profile of classical risk factors associated with cardiovascular disease, but also the association of non-classical risk factors, such as migraine headaches, neighborhood characteristics, social capital, and other psychosocial exposures with cardiovascular diseases. In terms of non-classical risk factors, subclinical thyroid diseases were included as a factor that may be associated not only with cardiovascular disease but also with chronic kidney disease, cognitive impairment, insulin resistance, metabolic syndrome, and mental health.

Most information about chronic diseases in the literature comes from large cohort studies in the United States and Western Europe, and there is little information about non-classical risk factors associated with cardiovascular disease in low-middle income countries like Brazil. Information about thyroid disease epidemiology in Brazil is scarce, but the studies that do exist suggest a high burden associated with thyroid disorders in the country (4–6) Therefore, data from the ELSA-Brasil is a very good opportunity to analyze, in a multicenter sample, the relationship between subclinical thyroid disorders and other conditions in a large Brazilian sample.

## Study design and objectives

The main objectives of the ELSA-Brasil are to study the incidence and progression of cardiovascular diseases and diabetes and associated factors such as biological, environmental, psychological, and social characteristics associated with these diseases. ELSA-Brasil is a multicenter prospective cohort study with investigation centers located in six cities in Brazil: Salvador, Vitória, Belo Horizonte, Rio de Janeiro, São Paulo, and Porto Alegre. All active and retired civil servants of these institutions aged between 35 to 74 years were eligible for the study. The exclusion criteria included current or recent pregnancy (at least four months before the first interview), intention to quit work in the near future, any communication impairment, or residence out the metropolitan area of the Research Center in each city. The baseline examinations were conducted from August 2008 to December 2010. After that, the participants were invited for the second visit, with further interviews and examinations, from August 2012 to December 2014. At the time of this report, the third visit was ongoing, which began in February 2017 and is planned to end in December 2018. In this review, we used only cross-sectional data from the baseline examination. Besides the visits, the study contacts all the participants by telephone once a year to ask about health outcomes, including any reason for hospitalization in the previous year, report of cardiovascular events, and cancer diagnosis (1).

Recruitment was performed according to sex, age, and socioeconomic status. The ELSA-Brasil sample is not representative of the country’s population, as the participants have higher levels of education and income status compared to the general Brazilian population. The applicability of the ELSA-Brasil estimates is supported by similarities in the prevalence of behavioral risk factors and chronic diseases, which were assessed using similar procedures to the Vigilância de Fatores de Risco e Proteção para Doenças Crônicas por Inquérito Telefônico (VIGITEL), a telephone-based behavioral risk factor survey that is conducted annually, with adults living in Brazil (7). However, the ELSA-Brasil was designed to evaluate associations between risk factors and cardiovascular diseases and diabetes.

The sample size was calculated considering an alpha value of 5%, statistical power of 80%, exposure prevalence of 20%, and relative risk of 2.0 for the incidence of myocardial infarction or diabetes, in approximately 6,400 subjects. This number was more than doubled to allow analyses by sex (1).

The baseline examination included a long questionnaire asking about sociodemographic risk factors, family history, morbidity, cardiovascular risk factors, psychosocial exposures, mental health, diet, and physical activity (8). Thyrotropin (TSH) and free thyroxine (FT4) were measured using a third-generation immunoenzymatic assay (Siemens, USA) in serum obtained from centrifuged venous blood samples after overnight fasting (9). In this review, reference range levels were 0.4–4.0 mIU/L for TSH and 10.3–24.45 pmol/L for FT4, similar to other Brazilian studies (10).

ELSA-Brasil participants were classified into five categories of thyroid function, according to TSH and FT4 levels and information about use of medication to treat thyroid disorders: clinical hyperthyroidism (low serum TSH and high FT4 levels or use of medication to treat hyperthyroidism), subclinical hyperthyroidism (low serum TSH, normal FT4 levels, and no use of drugs to treat thyroid diseases), euthyroidism (normal TSH and no use of thyroid drugs), subclinical hypothyroidism (high TSH levels, normal FT4 levels, and no use of drugs to treat thyroid diseases), and clinical hypothyroidism (high TSH and low FT4 levels, or use of levothyroxine to treat hypothyroidism). Accordingly, the definition of subclinical thyroid disease included only participants who did not use any drugs to treat thyroid disorders. Participants with overt thyroid disorders were excluded from the analyses. We also excluded participants using drugs that could interfere with thyroid function: amiodarone, carbamazepine, carbidopa, phenytoin, furosemide, haloperidol, heparin, interferon, levodopa, lithium, metoclopramide, propranolol, primidone, rifampicin, and valproic acid. The examination included a standard 12-lead electrocardiogram (ECG); measurement of pulse wave velocity (PWV) obtained using a validated noninvasive automatic device (Complior SP; Artech Medical, France) (11); heart rate variability to study the balance of the autonomic system determined from a 10 min ECG recording obtained in the supine position from DII derivation at 250 Hz (WinCardio; Micromed, Brazil) (12); carotid intima-media thickness (CIMT) using carotid ultrasonography of both common carotid arteries using a linear transducer of 7.5 MHz (Aplio XG, Toshiba, Japan) with axial resolution of approximately 0.10 mm (13,14); and measurement of coronary artery calcium (CAC) at the São Paulo Research Center as part of the baseline examination using a 64 detector computed tomography scanner (Brilliance 64; Philips Healthcare, The Netherlands). CAC is reported as Agatston units. CAC severity was further categorized with a cut-off at 100 points (<100 or ≥100) (15).

### Other measurements

Height and weight were measured using light clothes, and body mass index was calculated as weight divided by squared height in meters. Blood pressure (BP) measurements were taken using a validated Omron HEM 705CPINT (USA) oscillometric device. Three measurements were taken at one-min intervals. The mean of the two latter blood pressure measurements was considered as the participant’s casual blood pressure. Hypertension was defined as use of medication to treat hypertension, systolic blood pressure ≥140 mmHg, or diastolic blood pressure ≥90 mmHg. Diabetes was defined as a previous medical history of diabetes, use of medication to treat diabetes, fasting plasma glucose ≥6.99 mmol/L, 2 h plasma glucose ≥11.1 mmol/L, or HbA1C ≥6.5 (16). For measurement of fasting and post-load glucose, we used the hexokinase method (ADVIA 1200, Siemens), the immunoenzymatic assay for fasting and post-load insulin, and high-performance liquid chromatography (HPLC) (Bio-Rad Laboratories, USA) for glycated hemoglobin. Dyslipidemia was defined as LDL cholesterol ≥3.37 mmol/L or use of lipid-lowering medications. Low HDL-cholesterol was defined as HDL cholesterol <1.03 mmol/L in men and <1.29 mmol/L in women. Hypertriglyceridemia was defined as serum triglyceride levels ≥1.69 mmol/L. The enzymatic colorimetric assay (ADVIA 1200, Siemens) was used to measure total and HDL-cholesterol and triglycerides. LDL-cholesterol was calculated using the Friedewald equation, except for cases with elevated triglyceride levels (>4.52 mmol/L), when an enzymatic colorimetric assay was used (ADVIA 1200, Siemens). CKD-Epi was used to calculate the glomerular filtration rate (GFR) (17). Chronic kidney disease (CKD) was also defined as GFR <60 mL · min^−1^ · (1.73 m^2^)^−1^ and concomitance of albuminuria, according to the Kidney Disease Improving Global Outcomes (KDIGO) guidelines and previously published data on CKD in the ELSA-Brasil (Definition 2). Albuminuria was defined according to the albumin/creatinine ratio (18).

## Frequency of thyroid dysfunction and use of medication to treat thyroid diseases

Few studies have reported information about prevalence of thyroid diseases in Brazil (4–6) (Table 1). The population-based Sao Paulo Ageing & Health Study (SPAH) reported the prevalence of thyroid disorders in 1373 subjects aged 65 years or more. Frequency of thyroid diseases are higher in SPAH than in ELSA-Brasil especially for men. In the SPAH, 40% of women diagnosed with overt hypothyroidism were under treatment, compared to only 9% of men (5). In the ELSA-Brasil, a sample with more access to health services, we reported that the frequency of women receiving treatment for overt hypothyroidism was higher than that of men. However, the differences were smaller compared to the SPAH (97.3% of treatment in women *vs* 87.3% in men in the ELSA-Brasil). In the ELSA-Brasil, use of levothyroxine was very high, and was more frequent in women with higher mean monthly family income (Table 2).

**Table 1.** Prevalence of thyroid disorders by sex in large, population-based studies.

Study	All (%; 95%CI)	Men (%; 95%CI)	Women (%; 95%CI)
ELSA-Brasil (2015): 14,590 men and women 35–74 years from 6 capitals in Brazil (10)^a^			
Subclinical hyperthyroidism	1.3 (0-2.9)	0.5 (0-2.2)	0.8 (0-2.1)
Overt hyperthyroidism	0.7 (0-2.4)	0.3 (0-2)	0.4 (0-2.1)
Subclinical hypothyroidism	5.4 (3.8-7)	2.5 (0.9-4.1)	2.9 (1.3-4.5)
Overt hypothyroidism	7.4 (5.8-9)	1.2 (0-2.8)	6.2 (4.6-7.8)
Sao Paulo Ageing & Health Study (SPAH) (2013): 1373 men and women ≥ 65 years from poor area of São Paulo (5)^b^			
Subclinical hyperthyroidism	2.4 (1.6-3.2)	1.9 (0.7-3.0)	2.8 (1.6-3.9)
Overt hyperthyroidism	0.7 (0.2-1.1)	0.4 (0.01-0.9)	0.8 (0.2-1.5)
Subclinical hypothyroidism	6.5 (5.2-7.8)	6.1 (4.1-8.2)	6.7 (5.0-8.4)
Overt hypothyroidism	5.7 (4.5-6.9)	5.4 (3.5-7.3)	5.9 (4.3-7.5)
Population-based study in Rio de Janeiro: 1220 women ≥ 35 years from Rio de Janeiro (6)^c^			
Subclinical and overt hypothyroidism	12.3%		12.3%

^a^Adapted from reference 10; ^b^Adapted from reference 5; ^c^Adapted from reference 6.

**Table 2.** Studies published with data from ELSA-Brasil included in this review.

Study	Participants	Results	Conclusion
Olmos et al. (10)	14,590 participants: 12,437 (85.28%) with euthyroidism, 96 (0.7%) with overt hyperthyroidism, 193 (1.3%) with subclinical hyperthyroidism, 784 (5.4%) with subclinical hypothyroidism and 1080 (7.4%) with overt hypothyroidism.	Frequency of hypothyroidism treatment was higher in women, of Mixed race, and with high levels of education, and those with high net family incomes. After multivariate adjustment, levothyroxine use was associated with female sex (OR, 6.06, 95%CI, 3.19–11.49) and high net family income (OR, 3.23, 95%CI, 1.02–10.23). Frequency of hyperthyroidism treatment was higher in older than in younger individuals.	Sociodemographic factors strongly influenced the diagnosis and treatment of thyroid disorders, including the use of levothyroxine.

OR: odds ratio; 95%CI: 95% confidence interval.

In a population-sample of women in Rio de Janeiro, the prevalence of hypothyroidism was 12.3%, including overt and subclinical disease (6). White women presented a higher frequency of hypothyroidism than Mixed and Black women. In the ELSA-Brasil data, frequencies of subclinical and overt hypothyroidism were highest in White, intermediate in Mixed race, and lowest in Black participants (P<0.0001). After multivariate adjustment for sociodemographic variables, participants reporting Mixed or Black race presented a lower odds ratio (OR) for overt hypothyroidism using White race as reference (respectively, OR 0.73; 95%CI, 0.62–0.86 for Mixed, and OR, 0.53; 95%CI, 0.42–0.66 for Black). Regarding subclinical and clinical hyperthyroidism, White race was associated with higher frequency of hyperthyroidism compared to Mixed and Black races. In this case, after adjustment for the same variables, Black but not Mixed race was associated with overt hyperthyroidism (OR, 1.77; 95%CI, 1.03–3.03). These results agree with data from Sichieri et al. (6) in Rio de Janeiro that showed a lower frequency of hypothyroidism in Mixed race and Black subjects, and also with North-American results that showed an association between hyperthyroidism and Black race (19).

## Subclinical thyroid diseases: association with subclinical atherosclerosis, pulse wave velocity, and heart rate variability

### Thyroid function and carotid intima-media thickness

The ELSA-Brasil explored several measures of subclinical atherosclerosis as part of the study, including carotid intima-media thickness (CIMT) and coronary artery calcium (CAC). We analyzed the association between thyroid function and CIMT in 8,623 participants with no previous history of cardiovascular disease and a valid measurement of CIMT at baseline. Median CIMT was 0.60 mm (interquartile range (IQR)=0.53–0.70) in subclinical hypothyroidism *vs* 0.58 (0.51–0.67) in euthyroid subjects (P<0.0001) (Supplementary Table S1). After multivariate adjustment for sociodemographic and cardiovascular risk factors, subclinical hypothyroidism was associated with higher CIMT values using euthyroid subjects as reference (OR 1.30, 95%CI 1.06–1.59). No association was found between subclinical hyperthyroidism and CIMT (20).

Previous data on the relationship between thyroid function and CIMT showed conflicting results. Our findings were similar to a meta-analysis performed by Gao et al. (21) that showed a consistent association between subclinical hypothyroidism and CIMT. This review evaluated data from seven case-control studies and one cross-sectional study. Two other large cross-sectional studies (22,23) that were not considered in the meta-analysis of Gao et al. showed null associations between subclinical hypothyroidism and CIMT. Contrasting with our results, Völzke et al. (24), in a population-based study, reported an inverse association between TSH levels and CIMT. The only association found in our study was between CIMT with subclinical hypothyroidism (SCH), but never with subclinical hyperthyroidism.

### Thyroid function and CAC

In the ELSA-Brasil, the analysis of quintiles of TSH and CAC was restricted to 3836 participants of the Research Center of Sao Paulo who had undergone CAC as part of the baseline examination, and had no previous history of cardiovascular disease (Supplementary Table S1).

We found an association between low TSH levels (first quintile) and CAC with all men and women, even after multivariate adjustment for sociodemographic and cardiovascular risk factors (OR, 1.57; 95%CI, 1.05–2.35) using the third quintile as the reference. Restricting the analysis by sex, TSH in the first quintile was associated with CAC >100 Agatston units in men (OR, 1.72; 95%CI, 1.07–2.79). However, in women, we detected a U-shaped curve with an association of the first quintiles of TSH (OR, 3.31; 95%CI, 1.31–8.37 and of the fifth quintiles (OR, 3.29; 95%CI, 1.30–8.31) with CAC. It is important to note that restricting the analysis to TSH values below the reference showed similar results. Subjects with TSH within the range of subclinical hyperthyroidism and low-normal values (first quintile) had higher odds for CAC using the third quintile of TSH as reference (15).

Few published studies analyzed the association of subclinical thyroid diseases and CAC. A large South Korean study showed an inverse association among quartiles of TSH with CAC >0 in euthyroid participants using the first quartile as reference and TSH levels within the reference. Although there are many differences from the sample of that study and our sample, e.g., age and the distribution of cardiovascular risk factors, the results were similar, with an association between low TSH values and the presence of CAC (25). Another Korean study that also evaluated participants with TSH levels within the reference values did not report an association between TSH values and CAC (26). One study in Brazil reported an association between subclinical hypothyroidism and CAC >100 only in men older than 55 years, with a Framingham Risk Score ≥10% (27).

### Coronary heart disease using angiotomography

Data on the association between subclinical thyroid dysfunction and coronary artery disease (CAD) are scarce. In the ELSA-Brasil, we analyzed the association between thyroid function and CAD using a subsample of 767 participants who underwent complete coronary computed tomography angiography (CCTA) as part of the baseline examination (Supplementary Table S1). We included subjects with normal thyroid function, subclinical hypothyroidism, and subclinical hyperthyroidism. Logistic regression models evaluated quintiles of TSH and FT4 as independent variables, and the presence of CAD using segment involvement score (SIS) >4 and segment severity score (SSS) >4 of coronary arteries as dependent variables, adjusted for sociodemographic and cardiovascular risk factors. No association was found among TSH and FT4 quintiles and CAD prevalence, extent, or severity represented by SIS>4 and SSS>4. Restricting the analysis to euthyroid subjects did not alter the results. TSH and FT4 levels were not significantly associated with the presence, extent, or severity of CAD in a middle-aged healthy sample (28). The relatively small sample size may partially explain the results especially for subclinical hyperthyroidism.

Contrasting with our results, Park et al. (29) in the largest study to date evaluating the association of CAD using CCTA, found a strong association between subclinical hypothyroidism and CAD (adjusted OR, 2.13; 95%CI, 1.05 – 4.03). This was a retrospective analysis in South Korea that included 2,404 asymptomatic outpatients, mostly men, with 2% of participants with subclinical hypothyroidism. Similar to our study, they used CCTA for CAD diagnosis, which was defined as the presence of any degree of plaque detected in at least one segment. In contrast to our analysis, they included a higher frequency of men (97 *vs* 50.3% in our analysis), with higher 10-year CHD risk scores and higher TSH levels, which may partially explain the differing results. Our data contrasts with findings from a large meta-analysis by Collet et al. (30), which evaluated CAD events among subjects with subclinical hyperthyroidism in comparison with euthyroid ones. They included 52,674 participants from 10 cohort studies with available data about thyroid function, incident CAD, and mortality. However, none of those cohorts detected occult CAD using CCTA (2). Subclinical hypothyroidism was independently associated with CAD events (hazard ratio (HR), 1.21; 95%CI, 0.99–1.46), all-cause mortality (HR, 1.29; 95%CI, 1.02–1.62), and cardiovascular mortality (HR, 1.24, 95%CI, 1.06–1.46) (30). Another prospective study with a mean follow-up of around 3 years, 76 middle-aged subjects with subclinical hyperthyroidism and 1,062 euthyroid ones, all of them with type 2 diabetes mellitus, showed a higher incidence of CAD events with lower TSH levels (31). In our sample, only 0.5% (n=4) of the total number of subjects presented subclinical hyperthyroidism with TSH values lower than 0.1 mIU/L. Auer et al. used cineangiocoronariography to evaluate the relationship of subclinical thyroid disorders and coronary heart disease in 100 consecutive subjects, 90 euthyroid, 6 with subclinical hyperthyroidism, and 3 with subclinical hypothyroidism (59% men and mean of age 63.7 years). Contrasting with our results, they found an independent association between higher levels of TSH and severity of coronary arteries involvement, defined as two or three-vessel obstructive disease. However, they included a high frequency (35%) of double or triple-vessel disease, which may explain the difference from our results (32).

### Thyroid function and pulse wave velocity

Pulse wave velocity (PWV) measures arterial stiffness. In the ELSA-Brasil data for 8341 participants (5.6% of them with subclinical hypothyroidism), no association was detected between subclinical hypothyroidism and carotid-femoral pulse wave velocity (cfPWV) (Supplementary Table S1). The ELSA-Brasil is the largest study to investigate this association (33). A previous small study evaluated this association with positive results (34). One clinical trial reported lower PWV values after levothyroxine replacement (35). However, this information was not confirmed by another trial of the same research group (36). It is important to note that in the studies with positive results, Nagasaki et al. (35,36) measured brachial-ankle PWV compared to carotid-femoral PWV in the ELSA-Brasil.

### Thyroid function and heart rate variability

The association between subclinical thyroid dysfunction and autonomic modulation changes was analyzed using data from the ELSA-Brasil (Supplementary Table S1). The association between subclinical hyperthyroidism, subclinical hypothyroidism, and heart rate variability (HRV) was studied in 9270 subjects (median age 50 years; interquartile range: 44–56): 8,623 (93.0%) were classified as euthyroid, 136 (1.5%) as subclinical hyperthyroidism, and 511 (5.5%) as subclinical hypothyroidism. Compared to euthyroid subjects, those with subclinical hyperthyroidism presented a significantly higher heart rate (68.8 *vs* 66.5 for euthyroidism, P=0.007) and shorter R-R intervals (871.4 *vs* 901.6, P=0.007), although subclinical hyperthyroidism was associated with a lower standard deviation of NN interval (β: –0.070; 95%CI: –0.014 to –0.009) and low-frequency (β: –0.242, 95%CI: –0.426 to –0.058) compared to the euthyroid group. These differences lost significance after multivariate adjustment for confounders. No significant difference was found for HRV in subclinical hypothyroidism compared to euthyroid participants.

Several small studies have shown an association of subclinical hypothyroidism (37,38) and subclinical hyperthyroidism (39–41) with decreased heart rate variability. Compared to these small studies, our results did not show any association of HRV with subclinical hypothyroidism, and the association we found for subclinical hyperthyroidism lost significance after multivariate adjustment for sociodemographic and cardiovascular risk factors. The ELSA-Brasil is the largest study so far to evaluate HRV and thyroid function. The inclusion of apparently healthy individuals with lower levels of TSH than subjects selected from specialized outpatient clinics and the large sample that permitted adjustment for several confounders may help to explain differences in the results between the ELSA-Brasil and previous studies.

## Subclinical thyroid diseases: association with insulin resistance, metabolic syndrome, high-sensitivity C-reactive protein, and chronic kidney disease

### Insulin resistance and metabolic syndrome

In the ELSA-Brasil, after the exclusion of diabetics and individuals using cholesterol-lowering medications, we analyzed the relationship between TSH levels and insulin resistance and metabolic syndrome in a sample of 10,935 participants (54.3% women) (Supplementary Table S2). Homeostasis model insulin resistance (HOMA-IR) above the 75th percentile was defined as a surrogate for insulin resistance (HOMA-IR). Metabolic syndrome was diagnosed using NCEP ATP III criteria. We reported an association of quintiles of TSH and HOMA-IR above P75th percentile using the first quintile of TSH values as reference. Logistic regression models were built using HOMA-IR, with metabolic syndrome as the dependent variable and TSH quintiles as the independent variable (first quintile as reference). Subjects in the fifth TSH quintile presented an OR for association with insulin resistance of 1.86 (95%CI, 1.26–2.75), independent of sex. For the metabolic syndrome, subjects in the fifth quintile presented an OR of 1.21 (95%CI, 1.01–1.45). The analysis by sex remained positive only for men (OR 1.37; 95%CI 1.07–1.76). Restricting the analysis to TSH quintiles in the normal range did not affect the results (42).

Few studies have analyzed the association of thyroid function with insulin resistance and metabolic syndrome. For insulin resistance, most studies investigated small samples, consisting mainly of women, selected from tertiary care facilities, with conflicting positive (43,44) and negative (45,46) results. Furthermore, all these previous analyses categorized thyroid function as subclinical or clinical hypothyroidism. Compared to these small studies, the ELSA-Brasil included a high number of apparently healthy men and women, in a large sample of nondiabetics, more similar to the general population. Consistent with our findings, Garduão-Garcia, studying a sample of 3,148 Hispanics, reported an association between TSH values and HOMA-IR, with borderline significance (47). However, our data contrast with those of Lai et al. (48), who studied 1,534 participants in China and did not find any association between TSH in the normal range and HOMA-IR. Differences in study samples and the analysis strategy used could help explain some differences in results. For metabolic syndrome, most studies found a positive association with subclinical hypothyroidism for both sexes (49,50), although the study of Garduão-Garcia et al. (47) did not find any association. Although studies to evaluate the association between thyroid function and metabolic syndrome included a high number of participants, most of them also categorized thyroid function according to TSH and free thyroxine values, and restricted the evaluation to individuals with subclinical hypothyroidism compared with the present analysis that evaluated all ranges of TSH values categorized in quintiles. Our results for metabolic syndrome are not as clear as those for insulin resistance. We only found an association of TSH quintiles with metabolic syndrome in the overall sample, and only for men. However, our results for men are consistent with the findings of Lee et al. (51), who found an association between high-normal TSH concentration (still within the normal range) and metabolic syndrome.

### High-sensitivity C-reactive protein (hs-CRP)

A possible mechanism to explain the association of subclinical thyroid disorders and insulin resistance and metabolic syndrome is inflammation. Data from the ELSA-Brasil for the association of subclinical hypothyroidism and hs-CRP in 12,284 subjects (52.2% of women) with a median age of 50 years (interquartile range 45–57) reported that TSH was not associated with elevated hs-CRP, (OR, 1.11; 95%CI, 0.98–1.26) in a fully adjusted logistic model, also consistent with a linear model that analyzed the same relationship (β=0.024, P=0.145) (52) (Supplementary Table S2). Except for one large study that found a positive association between subclinical hypothyroidism and hs-CRP (53), most studies using different populations and analysis strategies reported no association between thyroid function and hs-CRP (54–56).

### Chronic kidney disease

Few studies have evaluated a possible relationship between TSH levels, glomerular filtration rate (GFR), and albumin/creatinine ratio in euthyroid subjects. We analyzed this association in a cross-sectional analysis in the ELSA-Brasil using GFR estimated by Chronic Kidney Disease Epidemiology Collaboration (CKD-Epi), with albuminuria/creatinine ratio as dependent variables and thyrotropin quartiles in individuals with euthyroidism and subclinical hypothyroidism as independent variables, adjusted for sociodemographic characteristics and diseases related to CKD (Supplementary Table S2). The definition of CKD was according to the Clinical Practice Guideline for the Evaluation and Management of Chronic Kidney Disease (KDIGO) (57).

We included 13,193 subjects with a median age of 51 years, IQR: 45–58, 51.8% women, and 5.9% with subclinical hypothyroidism. In the adjusted models, log-transformed TSH in euthyroid subjects was inversely and strongly associated with CKD (β = –2.181, 95%CI –2.714 to –1.648; P<0.0001) for glomerular filtration rate and for albuminuria/creatinine ratio (β =4.528; 1.190–7.865). Multivariate logistic models for euthyroid subjects showed an OR of 1.45 (95%CI, 1.15–1.83) for estimated GFR and of 1.95 (95%CI, 1.08–3.54) for albuminuria/creatinine ratio in the fourth TSH quartile, using the first quartile as the reference (62). Two large studies showed a higher prevalence of subclinical hypothyroidism among subjects with CKD, increasing with kidney dysfunction, using the abbreviated MDRD equation (58,59). In addition, similar to our findings, a large Norwegian population-based cross-sectional analysis found that TSH was negatively associated with GFR estimated using the MDRD equation, even in the reference range for TSH (60). Similarly, a prospective cohort study with 104,633 subjects and a mean follow-up of 3.5 years showed that high levels of TSH, even though still within the normal range, were modestly associated with an increased risk of incident CKD, defined as GFR <60 mL · min^−1^ · (1.73 m^2^)^−1^ using the CKD-Epi equation (61). Our study adds some new information to the previous published data. Some previous studies evaluating the relationship of thyroid disorders and CKD studied this association in a sample of individuals with hypothyroidism, including overt and subclinical disease (58,62). In our study, we restricted our analysis to subclinical hypothyroidism as in the Chonchol et al. (59) study. None of these previous studies have analyzed the relationship of thyroid disorders with albuminuria or with a combined endpoint of GFR <60 mL · min^−1^ · (1.73 m^2^) ^−1^ and albuminuria as in our study. Moreover, we studied this association in a different population, unlike previous studies conducted in the USA and Australia (58,59,62).

## Subclinical thyroid diseases: association with mental health and cognition

### Mental health

The ELSA-Brasil investigated the association between subclinical thyroid dysfunction and psychiatric disorders using baseline data in a cross-sectional analysis. The study included 12,437 participants with normal thyroid function (92.8%), 193 (1.4%) with subclinical hyperthyroidism, and 784 (5.8%) with subclinical hypothyroidism, totaling 13,414 participants (50.6% women) (63) (Supplementary Table S3). The mental health of the participants were assessed by trained health professionals, using the Clinical Interview Schedule - Revised (CIS-R) (64) and grouped according to the International Classification of Diseases 10 (ICD-10). After multivariate adjustment for possible confounders, we found a direct association between subclinical hyperthyroidism and panic disorder (OR, 2.55; 95%CI, 1.09–5.94), and an inverse association between subclinical hypothyroidism and generalized anxiety disorder (OR, 0.75; 95%CI, 0.59–0.96). However, both lost significance after correction for multiple comparisons (63).

Some previous studies with small samples detected changes in the hypothalamic-pituitary-thyroid axis in cases of panic disorder (65–67). Although the association between panic disorder and subclinical hyperthyroidism in our sample lost significance after correction for multiple comparisons, we have the possibility of analyzing the data prospectively in the near future. Interestingly, in another cohort in Brazil, we found an association between subclinical hyperthyroidism and dementia in a cross-sectional analysis of people ≥65 years old in a poor neighborhood of Sao Paulo (68). These findings, together with the results of this study, suggest that subclinical hyperthyroidism may be associated with psychiatric disorders and dementia in the Brazilian population.

Our analysis also detected a possible protective effect of subclinical hypothyroidism for anxiety disorder. Panicker et al. (69) reported a trend towards a low inverse relationship with anxiety: 19% less anxiety cases per mIU/l increase in TSH in men, and a trend towards less anxiety in women, both not using thyroxine in the HUNT Study. Our results for subclinical hypothyroidism suggest an association with depression, although not statistically significant. A previous population-based study in Rio de Janeiro, Brazil, found a positive association between subclinical hypothyroidism and depressive symptoms in women with TSH levels higher than 10 mIU/l. The ELSA-Brasil included a high number of men compared to the Guimarães study, which only included women (70). Three other studies in Brazil have reported a positive association between subclinical hypothyroidism and psychiatric symptoms and depression. However, all of them include only a small number of patients, mainly women referred to specialized clinics (71–73). Differences in study population and total sample size may partially explain the minor differences in the results.

### Cognitive impairment and dementia

The role of subtle thyroid alterations, such as subclinical thyroid disease and low/high serum TSH within the normal range, in cognitive decline is controversial. The aim of this study was to evaluate the association of serum TSH levels and subclinical thyroid dysfunction with performance in cognitive tests in a large sample of Brazilian middle-aged adults without overt thyroid disease. In this cross-sectional analysis, we excluded individuals aged 65 years and older, and with overt thyroid dysfunction (Supplementary Table S3). Cognition was evaluated using delayed word recall test, semantic verbal fluency test, and trail making test version B. The associations of cognitive tests performance with TSH tertiles (using the middle tertile as reference) and thyroid function were investigated using linear regression models, adjusted for an extensive set of possible confounders (sociodemographic characteristics, cardiovascular risk factors, and depression). The mean age of the 10,362 participants was 49.5±7.4 years with 52.3% women. After adjustment for confounders, the first TSH tertile was associated with worse performance on the trail making test (β=–0.05, 95%CI=–0.09; –0.01, P=0.017). When restricting the analysis to the 9,769 individuals with TSH within the normal range, the association between TSH and performance on the trail making test remained significant (β=–0.05, 95%CI=–0.09; –0.01, P=0.020) in the multiple linear regression. Subclinical thyroid disease was not associated with performance on cognitive tests. Our results showed that low TSH is associated with poorer performance in an executive function test in middle-aged adults without overt thyroid dysfunction (74).

Our study is similar to Wahlin et al. (75) who evaluated TSH levels and performance on cognitive tests and found that lower TSH was correlated with poorer performance in episodic memory, although TSH was not correlated with verbal fluency and visuospatial ability. A cross-sectional study in older adults without overt thyroid dysfunction and free of dementia found that participants in the lowest and second lowest quintiles of TSH had higher odds of cognitive impairment, assessed by the Mini-Mental State Examination, when compared to participants in the higher quintile of TSH (76). In our study, we found an association between low TSH levels and poorer performance in executive function, but no association with performance on episodic memory and verbal fluency. Other studies have also shown correlated results: low TSH associated with dementia or mild cognitive impairment (68,77), or subclinical hyperthyroidism associated with dementia (68,78) and poor performance in cognitive tests (79). However, some studies have shown the opposite: higher TSH levels related to dementia or poor cognitive performance (77,80), or no association at all between TSH and cognition (81,82). These discrepancies can be explained by the differences in sample, design, cognitive tests applied, selected covariates for adjustment, and outcomes between the studies. In addition, the association between TSH tertiles and the trail making test had only a small effect, which is expected in middle-aged subjects without overt thyroid disease and free of dementia and stroke at baseline. When considering the association between TSH levels within the normal range and cognition, studies also yield conflicting conclusions: some had results comparable to ours, showing an association between low TSH and dementia, mild cognitive impairment, or cognitive decline (68), but some did not find an association (81,77).

## Conclusions

Data from the ELSA-Brasil added important information about the association of subclinical thyroid disorders and subclinical atherosclerosis measured by carotid intima-media thickness and coronary artery calcium in a sample with different characteristics compared to previous published studies. We also detected an association with insulin resistance and metabolic syndrome, although no association was found for subclinical hypothyroidism with hs-CRP. Regarding chronic kidney disease, the ELSA-Brasil was the first study to include creatinine-albuminuria rate as part of the renal function evaluation. The association between subclinical hypothyroidism and chronic kidney disease in a cross-sectional analysis needs to be explored in a near-future prospective analysis, as this may be a consequence of reverse causation, as demonstrated by previous studies in other samples. A prospective association will be tested in the ELSA-Brasil in the near future. The association of subclinical hyperthyroidism with cognitive impairment corroborates with a previous study in Brazil. The association with psychiatric disorders needs to be tested in prospective analysis, since the number of psychiatric diagnoses at baseline was not enough to permit more complex analyses. Moreover, it is important to note that the use of L-thyroxine in this sample was higher in women and in participants in higher income strata, compared to others, and the frequency of treatment was lower in men with overt hypothyroidism compared to women. Thus, the importance of highlighting diagnosis and treatment of thyroid diseases in men is emphasized.

## Supplementary Material

Click here to view [pdf].
